# mTOR Blockade by Rapamycin in Spondyloarthritis: Impact on Inflammation and New Bone Formation *in vitro* and *in vivo*

**DOI:** 10.3389/fimmu.2019.02344

**Published:** 2020-02-27

**Authors:** Sijia Chen, Melissa N. van Tok, Véronique L. Knaup, Lianne Kraal, Désiree Pots, Lina Bartels, Ellen M. Gravallese, Joel D. Taurog, Marleen van de Sande, Leonie M. van Duivenvoorde, Dominique L. Baeten

**Affiliations:** ^1^Department of Experimental Immunology, Infection and Immunity Institute, Amsterdam University Medical Centers, Academic Medical Center, University of Amsterdam, Amsterdam, Netherlands; ^2^Department of Clinical Immunology and Rheumatology, Amsterdam Rheumatology & Immunology Center (ARC), Amsterdam University Medical Centers, Academic Medical Center, University of Amsterdam, Amsterdam, Netherlands; ^3^Division of Rheumatology, Inflammation, and Immunity, Brigham and Women's Hospital and Harvard Medical School, Boston, MA, United States; ^4^Internal Medicine, Rheumatic Diseases Division, UT Southwestern Medical Center, Dallas, TX, United States; ^5^UCB Pharma, Slough, United Kingdom

**Keywords:** spondyloarthtritis, mTOR, rapamycin, IL-17A, fibroblast-like synoviocytes, small molecule treatment, animal models, HLA-B27 tg rats

## Abstract

**Introduction:** Spondyloarthritis (SpA) is characterized by inflammation, articular bone erosions and pathologic new bone formation. Targeting TNFα or IL-17A with current available therapies reduces inflammation in SpA, however, treatment of the bone pathology in SpA remains an unmet clinical need. Activation of the mammalian target Of rapamycin (mTOR) promotes IL-17A expression and osteogenesis. Therefore, the inhibition of mTOR (with rapamycin) could be a promising therapeutic avenue in SpA.

**Objectives:** To investigate the effect of blocking mTOR on inflammation, bone erosions and new bone formation in SpA.

**Methods:** Peripheral blood mononuclear cells (PBMCs) from patients with SpA were stimulated with anti-CD3/CD28 in the presence or absence of rapamycin and the resulting cytokine expression was assessed. Fibroblast-like synoviocytes (FLS) from SpA patients were assessed for osteogenic differentiation potential in conditions with TNFα, IL-17A, or TNFα plus IL-17A, in the presence or absence of rapamycin. HLA-B27/Huβ2m transgenic rats were immunized with low dose heat-inactivated *Mycobacterium tuberculosis (M. tub)*, treated with 1.5 mg/kg rapamycin prophylactically or therapeutically and monitored for arthritis and spondylitis. Histology and mRNA analysis were performed after 5 weeks of treatment to assess inflammation and bone pathology.

**Results:** *In vitro* TNFα and IL-17A protein production by SpA PBMCs was inhibited in the presence of rapamycin. Rapamycin also inhibited osteogenic differentiation of human SpA FLS. *Ex vivo* analysis of SpA synovial biopsies indicated activation of the mTOR pathway in the synovial tissue of SpA patients. *In vivo*, prophylactic treatment of HLA-B27/Huβ2m transgenic rats with rapamycin significantly inhibited the development and severity of inflammation in peripheral joints and spine (arthritis and spondylitis), with histological evidence of reduced bone erosions and new bone formation around peripheral joints. In addition, therapeutic treatment with rapamycin significantly decreased severity of arthritis and spondylitis, with peripheral joint histology showing reduced inflammation, bone erosions and new bone formation. *IL-17A* mRNA expression was decreased in the metacarpophalangeal joints after rapamycin treatment.

**Conclusion:** mTOR blockade inhibits IL-17A and TNFα production by PBMCs, and osteogenic differentiation of FLS from patients with SpA *in vitro*. In the HLA-B27 transgenic rat model of SpA, rapamycin inhibits arthritis and spondylitis development and severity, reduces articular bone erosions, decreases pathologic new bone formation and suppresses IL-17A expression. These results may support efforts to evaluate the efficacy of targeting the mTOR pathway in SpA patients.

## Introduction

Spondyloarthritis (SpA) is the second most prevalent form of chronic inflammatory arthritis. The hallmarks of SpA are joint inflammation, articular bone erosions and pathologic new bone formation (1). Tumor necrosis factor-α (TNFα) and Interleukin-17A (IL-17A) are key disease-modulating cytokines in SpA (1–4). Currently, one third of patients do not respond to available therapy and only 20% of patients achieve remission. Loss of therapeutic efficacy can occur over time and anti-TNFα therapy does not reduce bone formation in the advanced stages of SpA (5–8). Although anti-IL-17A therapy has been demonstrated to reduce bone formation the *M.tub-*induced HLA-B27 transgenic rat model (HLA-B27 tg rats) (9), a beneficial effect of anti-IL-17A therapy on human SpA bone pathology is not well-understood and remains to be formally established (3, 5). Thus, there is an unmet clinical need to find therapies targeting both inflammation and pathologic bone formation in SpA.

The etiology of the new bone formation in SpA remains unclear (1, 10). We and others (9, 11, 12) have previously demonstrated that fibroblast-like synoviocytes (FLS) isolated from the synovial tissue may act as bone precursor cells and differentiate *in vitro* to osteoblast-like-cells. Osteoblasts are the bone-forming cells responsible for bone matrix and bone mineralization (10). It has also been demonstrated that TNFα and IL-17A can accelerate osteogenic differentiation of FLS cells *in vitro* (9, 12).

The mammalian target of rapamycin (mTOR) has been demonstrated to play an important role in inflammation. For instance, mTOR, can be blocked using rapamycin, a small molecular drug that has been applied clinically to prevent graft rejection in kidney transplantation (13–15). mTOR has been demonstrated to activate T cells and regulate RORγ translocation in murine cells to induce IL-17A expression (16, 17). Blocking mTOR reduces the percentage of Th17 cells in an animal model of colitis (18). RNA sequencing of inflamed synovial tissue from patients with SpA demonstrated the expression of the PI3K-Akt-mTOR pathway (Chen and Ross et al. under review).

In addition to modifying inflammation, mTOR signaling is downstream of bone anabolic pathways and promotes osteoblastic maturation and mineralization (19).

We sought to examine the effect of mTOR blockade with rapamycin on inflammation as well as new bone formation in the pathobiological context of SpA. Since mTOR pathway regulates the expression of the disease modulating cytokine IL-17A and promotes osteogenic differentiation we hypothesized that treatment with rapamycin may modify both inflammation and pathologic bone formation in SpA pathogenesis. Initially, we determined the effect of rapamycin *in vitro* on primary human SpA cells. Specifically, we investigated if rapamycin could inhibit the production of cytokines by SpA peripheral blood mononuclear cells (PBMCs) and if rapamycin could reduce the rate of human SpA FLS to differentiate to osteoblast-like-cells. Next, we confirmed the activation of mTOR pathway in SpA synovitis. In addition, we determined the prophylactic and therapeutic treatment effect of rapamycin in the *M.tub*-induced HLA-B27 transgenic rat model (HLA-B27 tg rats), an experimental model of SpA (20–22). In the HLA-B27 tg rats, we assessed whether rapamycin would reduce the incidence and severity in inflammation of peripheral joints and spine (arthritis and spondylitis), bone erosions and pathologic new bone formation *in vivo*.

## Materials and Methods

### Human Cells and Tissue

Patient material was obtained from spondyloarthritis (SpA) and rheumatoid arthritis (RA) patients. The SpA patients included in this study fulfilled the Assessment of Spondyloarthritis International Society (ASAS) criteria for peripheral SpA (23). The RA patients were included according to the American College of Rheumatology classification criteria (24). All patients provided written informed consent before enrollment in the study. This study was approved by the Ethics Committee of the Amsterdam University Medical Center, University of Amsterdam, the Netherlands.

### *In vitro* Stimulation of Human PBMCs and SFMCs

Primary human peripheral blood mononuclear cells (PBMCs) were obtained from healthy donors (*n* = 3) and SpA patients (*n* = 6), and synovial fluid mononuclear cells (SFMCs) were obtained from inflamed knee joints from SpA patients (*n* = 2). At the time of inclusion, SpA patients had not taken biologic agents for at least 3 months. PBMCs and SFMCs were isolated by density gradient centrifugation on Lymphoprep (Nycomed).

PBMCs and SFMCs were pre-incubated with vehicle (0.001% DMSO) or rapamycin in IMDM (Lonza) for 30 min and stimulated with anti-CD3 (clone 1XE, 1: 1,000, Sanquin) and anti-CD28 (clone 15E8, 2 μg/ml, Sanquin) for 48 h. Cytokines were measured in supernatants by ELISA (IL-17A and TNFα, eBioscience) according to the manufacturer's recommendations. Counting of viable PBMCs was performed by flow cytometry on an LSR Fortessa X-20 instrument (BD). Exclusion of DAPI (10 nM, 46-Diamidino-2-Phenylindole, Dihydrochloride; Sigma) and PI (3 μM, Propidium iodide; Sigma) was used to indicate cell viability. Accudrop Beads (BD) were used as the counting standard. Per sample, 10,000 beads were added and PBMCs were counted during the acquisition of 6,000 beads.

### *In vitro* Osteogenic Differentiation of SpA Fibroblast-Like Synoviocytes (FLS)

Primary human SpA FLS (*n* = 11) were obtained from synovial tissue biopsies according to standardized protocol (9, 25). FLS viability was assessed in the presence of vehicle (0.00005%DMSO) or rapamycin (5 nM) with WST-1 assay (Roche) and Trypan Blue 0.4% solution (Gibco) (26). For *in vitro* differentiation, SpA FLS (passage 3–8) were cultured in Xvivo Stempro medium (R&D systems) with 50 μM ascorbic acid and 10 mM β-glycophosphate, supplemented with TNFα (1 ng/ml), IL-17A (50 ng/ml), or both. Media was refreshed twice weekly. Cells were fixed with 4% formaldehyde at days 7, 14, and 21 of differentiation. Alkaline phosphatase (ALP) staining was performed at days 7 and 14. Alizarin red (2%) staining was performed at day 21 for the osteogenic conditions without cytokines and at day 14 for conditions supplemented with cytokines, consistent with prior published protocols (9). The percentage of ALP staining and alizarin red staining in the wells were scored semi-quantitively by 2 observers, as described previously (9).

### Immunofluorescence

Frozen synovial tissue sections (*n* = 20 from SpA patients and *n* = 20 from RA patients) were fixed and blocked with 10% serum. Staining with isotypes or primary antibodies was performed overnight at 4°C, followed by incubation with Alexa Fluor 488/Alexa Fluor 594-conjugated secondary antibodies for 30 min at room temperature. Antibodies used: monoclonal rabbit IgG anti-human phospho-S6 (pS6, ser235/236, clone D57.2.2E, Cell signaling); monoclonal mouse IgG1 anti-human CD3 (clone UCH-T1; Thermo Scientific Pierce); and monoclonal mouse IgG1 anti-human CD45 (HI30; Biolegend) at a concentration of 5 μg/ml. Slides were mounted with Prolong Gold with DAPI (Thermo Fisher). Pictures were taken on an epifluorescence imaging microscope (Leica) and analyzed using ImageJ (1.50i) software. The quantity of pS6 staining was scored on a 3-point semiquantitative scale by two independent observers, who were blinded for the diagnosis of the patients, according to standardized methods (9, 27–29).

### Animals

In order to generate *M.tub-*induced HLA-B27/Huβ2m transgenic rats (HLA-B27 tg rats), the Tg(HLA-B*2705, B2M)21-3Reh and Tg(B2M)283-2Reh Lewis rat lines (30) were bred and housed at the animal research institute of AMC. F1 (21–3 × 283–2) male and female rats were used for experiments. Animal experiments were approved by the Amsterdam University Medical Center (AUMC) Animal Care and Use Committee.

Male rats were orchiectomized to prevent epididymo-orchitis (31), as described previously (22). To synchronize disease onset, 6-week-old, HLA-B27/Huβ2m transgenic rats were immunized with heat-inactivated *Mycobacterium tuberculosis (M. tub)* (Difco, Detroit, MI, USA) in 100 μl Incomplete Freund's Adjuvant (IFA) (Chondrex, Redmond, WA, USA) as described previously (21, 22).

### *In vivo* Preventive and Therapeutic Treatment With Rapamycin

Rats were treated with 1.5 mg/kg rapamycin or vehicle intra-peritoneally, three times per week for 5 weeks. The prophylactic treatment (*n* = 13 vehicle vs. *n* = 11 rapamycin) started 1 week post-immunization with *M. tub*, which is ~1–2 weeks before the onset of arthritis and spondylitis. The therapeutic treatment (*n* = 5 vehicle vs. *n* = 4 rapamycin) started 1 week after 50% of the animals developed arthritis. Vehicle-treated rats were caged separately from rapamycin treated rats.

### Clinical Measurement of Arthritis and Spondylitis

The HLA-B27 tg rats were monitored for arthritis and spondylitis incidence and severity, as described previously (9, 21, 22). Clinical measurements were performed by an observer blinded for treatment, and included weight, macroscopic severity scores for arthritis (0–12) and spondylitis (0–3), and hind paw swelling measured by plethysmometry. For the severity analysis, cumulative clinical scores of all limbs were calculated. For plethysmometry, the change in swelling in cm^3^ was normalized to the measurement on the day before the disease onset as observed clinically (prophylactic experiment) or to the day of treatment start (therapeutic treatment).

### Histology

Rats were sacrificed after 5 weeks of treatment. Hind paws (peripheral joints) and the tail (axial joints) were isolated and fixed in 10% formalin, decalcified in Osteosoft (Merck) and embedded in paraffn. For hematoxylin and eosin (H&E) or Safranin O/Fast Green staining, 5 μm sections were stained and scored by two observers blinded to the identities of the treatment groups. Semi-quantitative scoring was performed for inflammation, bone erosions, periosteal new bone formation and hypertrophic chondrocytes (enchondral new bone formation) as described previously (21, 22). Images were obtained with a light microscope (Leica).

### Gene Expression Analysis

Metacarpophalangeal (MCP) joints were homogenized in TRIzol and further processed for RNA isolation on columns (RNAeasy mini columns, Qiagen) according to manufacturer's protocol.

qPCRs were performed with SYBR green primers (Life technologies) for *IL-17A, IL-17F, IL-22, TNF*α*, IL-23, RORC, IFN*γ*, IL-4*, with *GAPDH* as the reference gene. Data were represented as relative fold-changes [according to the 2^−ddCt^ method (32)] to one reference control sample. GAPDH expression was detected in all samples with <22 Ct cycles. When a gene was not detectable, Ct 40 was used for the calculation of the relative fold.

### Statistics

Graphpad Prism 7 was used to perform statistical analysis. For all normally-distributed continuous data, the one-way ANOVA was performed with multiple comparison adjustments according to Bonferroni. Survival curves were analyzed for arthritis and spondylitis incidence and compared with the Log-Rank (Mantel-Cox) test. The Area Under the Curve (AUC) was calculated for clinical scores and hind paw swelling and analyzed with a Mann–Whitney *U*-test. For non-normally distributed data and nominal data, Mann–Whitney *U*-test was used.

## Results

### Rapamycin Inhibits IL-17A and TNFα Protein Production by Human PBMCs From SpA Patients

**Figure 1:**
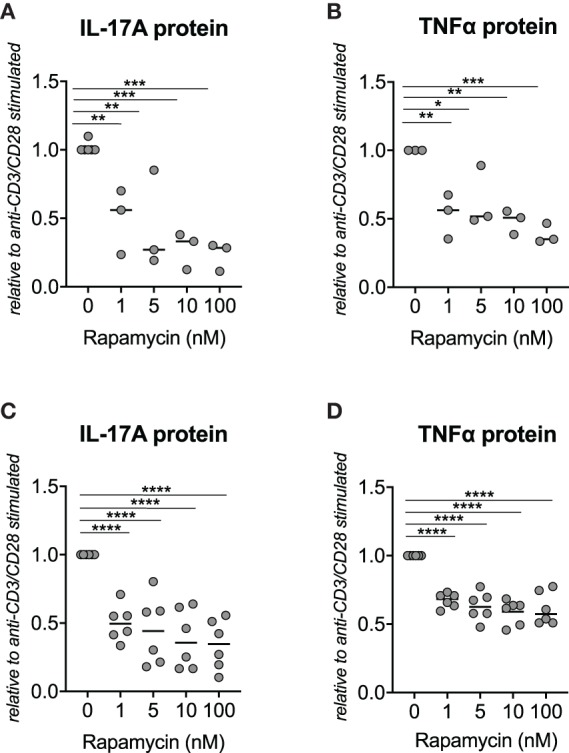
Rapamycin reduces IL-17A and TNFα protein production by PBMCs from Spondyloarthritis (SpA) patients *in vitro*. **(A)** Rapamycin reduces IL-17A (*n* = 3) and **(B)** TNFα (*n* = 3) protein secretion by *in vitro* stimulated PBMCs from healthy donors. **(C)** Rapamycin inhibits production of IL-17A (*n* = 6) and **(D)** TNFα (n = 6) by PBMCs from patients with SpA. ****p < 0.0001, ***p < 0.001, **p < 0.01, and *p < 0.05.

As mTOR activation has been demonstrated to induce IL-17A expression in murine T cells (16), we hypothesized that rapamycin treatment would inhibit IL-17A production in human PBMCs. We therefore first examined the effect of rapamycin (0, 1, 5, 10, 100 nM) in healthy donor PBMCs stimulated with anti-CD3/CD28 antibodies. Rapamycin significantly reduced IL-17A and TNFα protein secretion by *in vitro* stimulated healthy donor PBMCs, with a reduction of 51.8% for IL-17A and 47.0% for TNFα at 1 nM, respectively (Figures 1A,B). Rapamycin did not induce cell death over the culture period of 48 h as measured by DAPI and PI staining by flow cytometry (Supplemental Figure 1).

Similarly, rapamycin significantly reduced IL-17A and TNFα production by human SpA PBMC: 1 nM of rapamycin induced a 50.0% reduction of IL-17A and 32.7% reduction of TNFα production (Figures 1C,D). A similar but non-significant trend was also observed when human SpA SFMCs were treated *in vitro* with rapamycin (Supplemental Figures 2A,B).

### Rapamycin Reduces Osteogenic Differentiation of Human SpA Fibroblast-Like Synoviocytes (FLS)

In bone precursor cells, the mTOR pathway has also been reported to promote osteogenesis through regulation of bone anabolic pathways (19). To study a potential direct effect of mTOR inhibition by rapamycin on osteoblastic differentiation within the inflammatory context of SpA, we performed *in vitro* osteogenic differentiation assays with human FLS, in the presence and absence of key proinflammatory cytokines IL-17A and TNFα (9). During the differentiation process, we stained the cells for Alkaline phosphatase (ALP) and for mineralization with alizarin red. ALP is expressed early in the osteogenic differentiation process and mineralization is a characteristic of matured osteoblasts.

**Figure 2:**
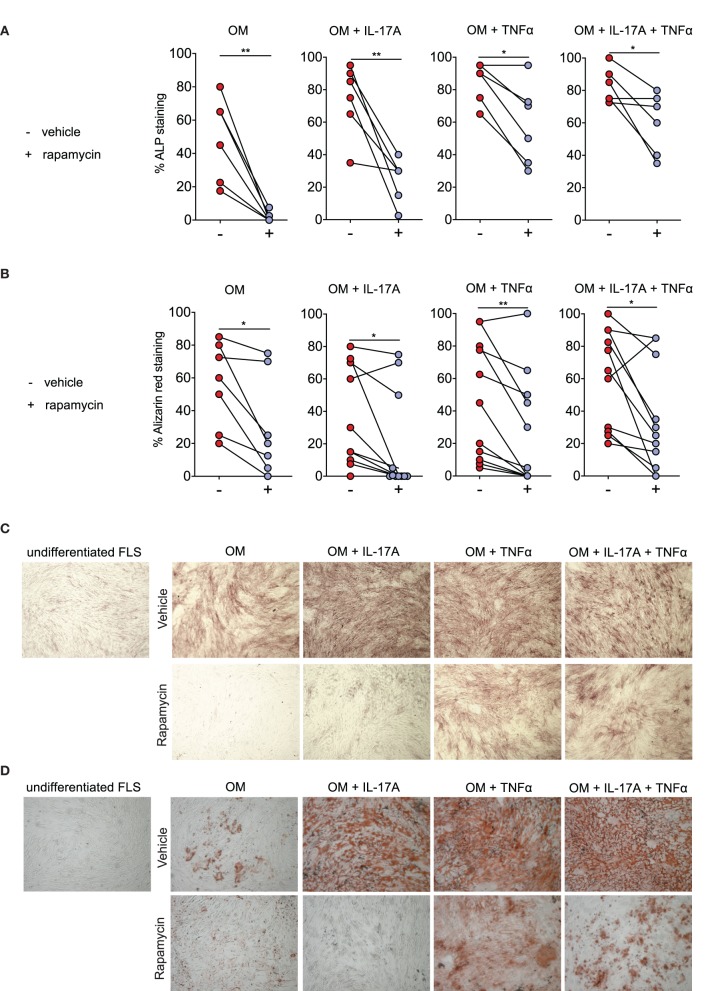
Rapamycin reduces Spondyloarthritis (SpA) fibroblast-like synoviocytes (FLS) osteogenesis. **(A)** Rapamycin reduces osteogenic differentiation as evidenced by percentage of Alkaline phosphatase (ALP) staining at day 14 (*n* = 6), as well as by **(B)** percentage of alizarin red staining at at day 21 for OM (n = 7), and day 14 for the conditions with cytokines (n = 11). **(C)** Representative pictures of ALP staining and **(D)** alizarin red staining. OM: osteogenic media. **p < 0.01 and *p < 0.05.

A dose-response experiment showed that ≥5 nM rapamycin consistently reduced osteogenic differentiation of human SpA FLS *in vitro*, as evidenced by alizarin red staining (data not shown). There was no increase in cell death after rapamycin treatment, as demonstrated by WST-1 assay and by Trypan Blue staining (Supplemental Figure 3). We confirmed this effect in FLS cells from additional SpA patients, finding that rapamycin (5 nM) treatment significantly reduced ALP staining, a marker for active (pre-) osteoblasts (Figure 2A), and alzarin red staining, a marker for mineralization (Figure 2B).

Rapamycin (5 nM) also significantly reduced osteogenic differentiation of human FLS in the presence of IL-17A and/or TNFα (Figures 2A–D). Consistent with prior report (9), IL-17A and TNFα accelerated human FLS osteoblastic differentiation as demonstrated by increased ALP staining (Figure 2A) and alizarin red staining (Figure 2B). Representative ALP (Figure 2C) and alizarin red (Figure 2D) pictures from one SpA FLS line are shown. These data indicate that rapamycin can reduce the osteoblastic differentiation rate of human SpA FLS *in vitro*.

### mTOR Pathway Is Activated in Inflamed Synovial Tissues From Patients With SpA

**Figure 3:**
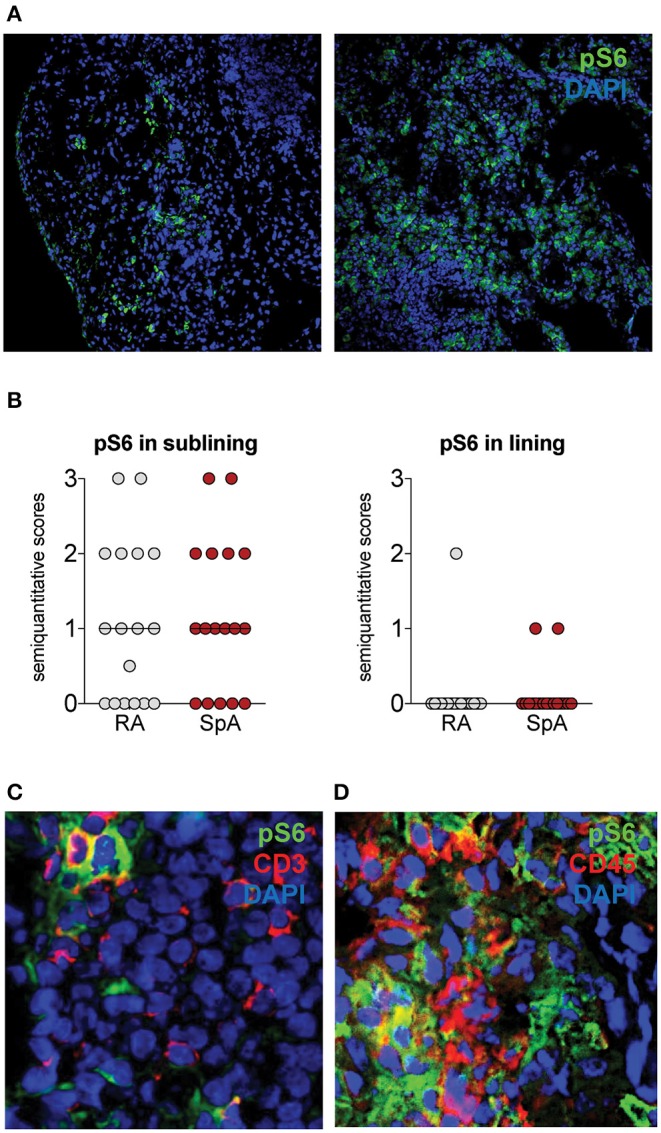
pS6 is expressed in Spondyloarthritis (SpA) synovitis. **(A)** pS6 is mainly expressed in the sublining of the synovial tissue. Representative pictures from 2 SpA patients are shown. **(B)** Semiquantitative scoring of Ps6 in Rheumatoid Arthritis (RA, *n* = 20) and SpA (*n* = 20) synovial tissue. **(C)** pS6 partially colocalizes with CD3 by double immunofluorescence. **(D)** pS6 partially colocalizes with CD45.

We next assessed whether the mTOR pathway was activated in SpA synovitis. As phospho-S6 Ribosomal Protein (pS6) is a well-characterized downstream target of mTOR and indicates activation of the mTOR pathway, we stained for the presence of pS6 in inflamed SpA synovial tissue. Representative images are shown from 2 patients (Figure 3A). The majority of pS6 positive cells was observed in the sublining of the synovial tissue (Figures 3A,B). In addition to SpA synovial tissue, we also stained synovial tissue from rheumatoid arthritis (RA) patients, as the presence of pS6 and the expression of mTOR pathway have previously been described in RA synovitis (33).

pS6 levels are similar in SpA and RA synovial tissue groups as demonstrated by semiquantitative scoring (Figure 3B). In SpA synovial tissue, we observed colocalization of pS6 with CD3, a marker for T cells (Figure 3C). T cells from the synovial tissue have been demonstrated to express IL-17A (34). pS6 also stained CD45-negative cells (Figure 3D), indicating that the mTOR pathway is also activated in non-hematopoietic, stromal cells in SpA synovitis.

### Prophylactic Treatment With Rapamycin Reduces Experimental Spondyloarthritis and New Bone Formation *in vivo*

**Figure 4:**
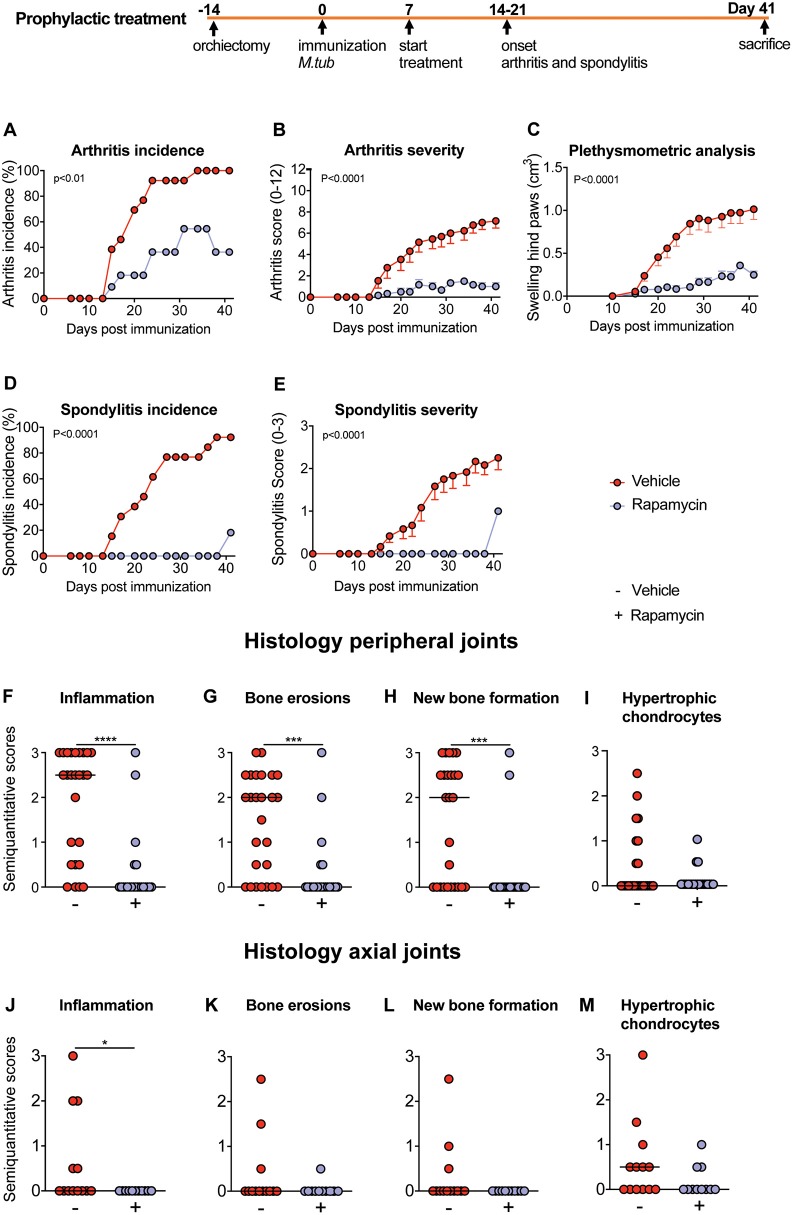
Preventive treatment with rapamycin reduces experimental spondylarthritis *in vivo*. **(A)** Arthritis incidence. **(B)** Arthritis severity. **(C)** Plethysmometric analysis of the hind paws. **(D)** Spondylitis incidence and **(E)** spondylitis severity of vehicle (*n* = 13) and rapamycin treatment group (*n* = 11). **(F–I)** Semiquantitative scoring of inflammation, bone erosions and new bone formation in peripheral joint histology and in **(J–M)** axial histology. Mean ± SEM were presented in **(B,C,E)**. Median was depicted in **(F–M)**. *M.tub: Mycobacterium tuberculosis*. ****p < 0.0001, ***p < 0.001, and *p < 0.05.

Next, we tested the effcacy of rapamycin treatment in HLA-B27 tg rats, an experimental model of spondyloarthritis (9, 22, 35). In a prophylactic setting, 100% (Figure 4A) and 92% (Figure 4D) of animals developed arthritis and spondylitis, respectively, in the vehicle control group. Prophylactic rapamycin treatment significantly decreased the incidence of arthritis (36%, Figure 4A) and spondylitis (18%, Figure 4D). Moreover, arthritis severity (0.5 vs. 7.15 on a 0–12 scale, Figure 4B) and spondylitis severity (0.2 vs. 2.1 on a 0–3 scale, Figure 4E) were significantly reduced in the rapamycin vs. vehicle group. Plethysmometric analysis confirmed the significantly reduced swelling in the hind paws in the rapamycin treatment group (Figure 4C). Histological analysis of peripheral joints confirmed these clinical findings, demonstrating significantly reduced inflammation, bone and cartilage erosions, and new periosteal bone formation (Figures 4F–H), and a similar trend for the presence of hypertrophic chondrocytes (Figure 4I). The animals treated with rapamycin also had reduced inflammation in the spine (Figure 4J). Similar, but not significant, trends were observed for bone erosions, new bone formation and the presence of hypertrophic chondrocytes in the spine (Figures 4K–M). Prophylactic treatment data were pooled from two independent experiments.

### Therapeutic Treatment With Rapamycin Attenuates Experimental Spondyloarthritis *in vivo*

**Figure 5:**
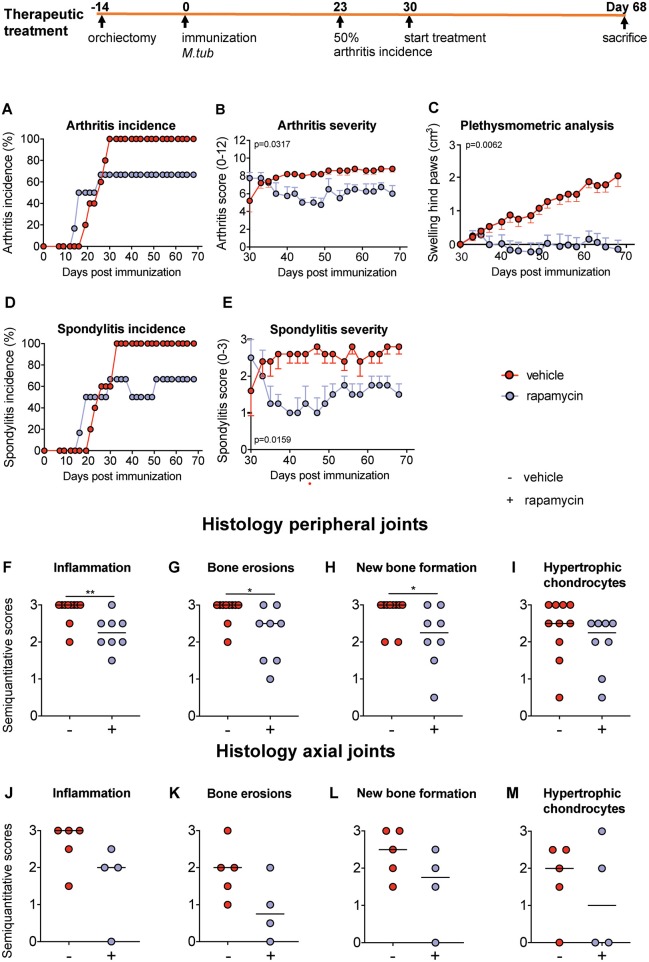
Therapeutic treatment with rapamycin attenuates experimental spondyloarthritis *in vivo*. **(A)** The incidence of arthritis and **(D)** spondylitis are similar in the vehicle (*n* = 5) and rapamycin (*n* = 4) group at treatment initiation. In the diseased animals, treatment with rapamycin **(B)** diminished arthritis severity and **(E)** spondylitis severity. **(C)** Plethysmometric analysis of hindpaw swelling. **(F–I)** Semiquantitative scoring of inflammation, bone erosions and new bone formation in peripheral joint histology and in **(J–M)** axial histology. Mean ± SEM were presented in **(B,C,E)**. Median was depicted in **(F–M)**. *M.tub: Mycobacterium tuberculosis*. **p < 0.01 and *p < 0.05.

**Figure 6:**
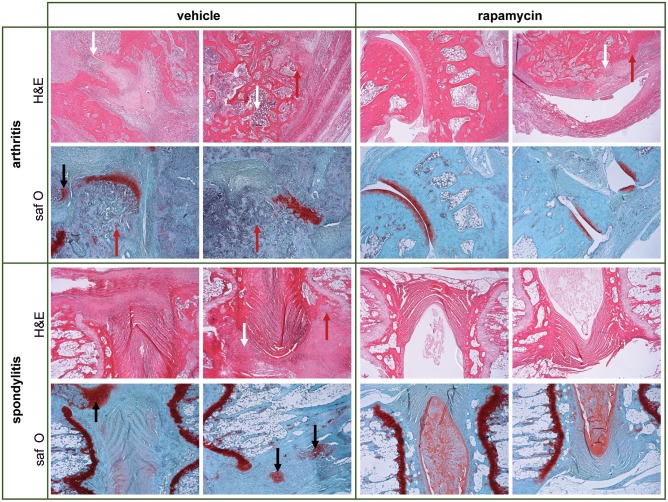
Therapeutic treatment with rapamycin reduces inflammation, bone erosions and new bone formed in experimental spondyloarthritis. Representative hematoxylin and eosin (H&E) & Safranin O/Fast Green (saf O) pictures are shown for vehicle and rapamycin treated rats, to demonstrate aspects of pathology in the model. There is deformity of the joint anatomy with aspects of inflammation (white arrows), destruction/erosions (red arrows), and new bone formation (black arrows) in peripheral and axial joints. The quantification of the pathology is provided in Figures 5F–M for the therapeutic experiment (and in Figures 4F–M for the prophylactic experiment).

We next assessed the effect of rapamycin on inflammation and bone pathology in a therapeutic setting. Treatment was started 1 week after 50% arthritis incidence (Day 30 after immunization with *M. tub*; Figure 5). In the vehicle group, the incidence of arthritis and spondylitis continued to increase to 100% within 2 weeks after treatment initiation, whereas the incidence of both arthritis and spondylitis completely plateaued once rapamycin treatment was initiated (Figures 5A,D). Furthermore, therapeutic treatment with rapamycin significantly diminished arthritis severity (6 vs. 8.8; Figure 5B) and spondylitis severity (1.5 vs. 2.8; Figure 5E). In agreement with the clinical scores, plethysmometric analysis demonstrated a significant reduction in hind paw swelling in the rapamycin group compared to vehicle controls (Figure 5C). Histological analysis of peripheral joint tissues obtained 5 weeks after initiation of treatment confirmed the impact of rapamycin treatment on inflammation (Figure 5F), bone erosions (Figure 5G), and new periosteal bone formation (Figure 5H). A similar, but not significant trend was observed for hypertrophic chondrocytes in peripheral joints (Figure 5I). Histological analysis of the spine revealed similar trends (Figures 5J–M). Representative H&E and Safranin O staining of the peripheral joints and spine are shown in Figure 6.

### Rapamycin Treatment Reduced IL-17A Expression in Inflamed Joints

**Figure 7:**
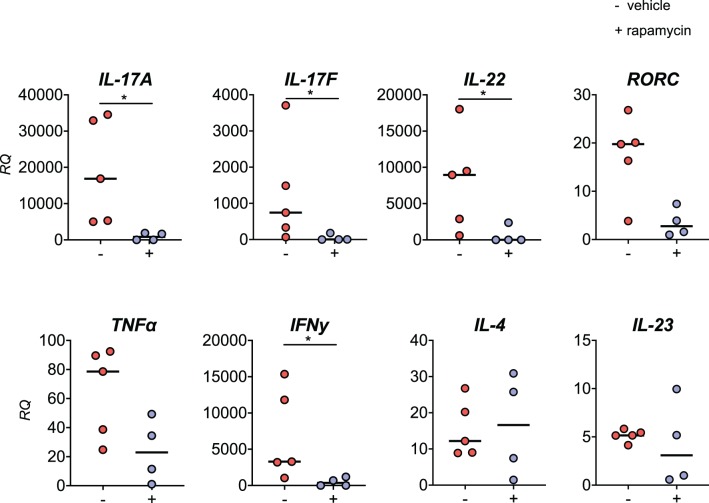
Rapamycin treatment reduces IL-17A expression in inflamed joints. In the metacarpophalangeal (MCP) joints from HLA-B27 tg rats after the therapeutic treatment, mRNA expression of *IL-17A, IL-17F, IL-22*, and *IFN*γ are reduced in the rapamycin group. RQ, relative quantity. *p < 0.05.

We demonstrated that rapamycin inhibits IL-17A and TNFα production by human SpA PBMCs *in vitro*. To test the *in vivo* effcacy of rapamycin in the HLA-B27 tg rat model, we assessed the mRNA expression of key inflammatory cytokines such as IL-17A and TNFα in metacarpophalangeal (MCP) joints after therapeutic treatment with rapamycin. mRNA expression of *IL-17A, IL-17F*, and *IL-22* was significantly reduced in the MCP joints from the rapamycin vs. vehicle treatment group (Figure 7). This was paralleled by a trend toward reduction of the IL-17 master transcription factor *RORC*, but not the upstream Th17 differentiation cytokine *IL-23*. Also *IFN*γ mRNA expression was significantly reduced. There was a decreasing trend observed for *TNF*α, whereas the prototypical Th2 cytokine *IL-4* was not modified.

## Discussion

We demonstrate here for the first time that targeting mTOR by rapamycin, prophylactically and therapeutically, reduces the incidence and severity of arthritis and spondylitis. Histology confirmed reduced inflammation, bone erosions and new periosteal bone formation in the peripheral joints with similar trends observed in the spine. These findings were supported by data we obtained *in vitro* in human SpA cells and from human synovial tissue *ex vivo*: rapamycin attenuated inflammatory cytokine production by human SpA PBMCs; rapamycin reduced human SpA fibroblast-like synoviocytes (FLS) osteogenesis rate, both in the presence and absence of TNFα and IL-17A; and the mTOR pathway is activated in human SpA synovitis.

Taken together, these data suggest that mTOR blockade by rapamycin attenuates inflammation, bone remodeling and new periosteal bone formation, which are all hallmarks of SpA pathogenesis. mTOR might be a promising therapeutic target in SpA patients, especially considering the effcacy of rapamycin in reducing new bone formation and bone erosions *in vivo*. This would address an important unmet clinical need in SpA patients to target bone pathology (1, 5).

Two potential explanations for the inhibitory effect of rapamycin on new bone formation in the HLA-B27 tg model are: it may either be the result of rapamycin reducing expression of the cytokine IL-17A, or a direct inhibitory effect of rapamycin on bone precursor cells. We have previously demonstrated that IL-17A promotes pathologic bone processes in the HLA-B27 tg rats and shown that IL-17A accelerates osteogenic differentiation of human SpA FLS *in vitro* (9). In line with these findings, others have reported that IL-17A accelerates osteogenic differentiation of FLS cells from reumatoid arthritis (RA) and osteoarthritis (OA) patients (12). We now demonstrate that FLS osteogenesis can be inhibited by rapamycin treatment, independently of IL-17A and TNFα cytokines. Rapamycin treatment in SpA PBMCs *in vitro* also inhibits production of IL-17A and TNFα. In addition to TNFα (1) and IL-17A (2, 3), IL-17F has recently been demonstrated to play an essential proinflammatory role in SpA pathogenesis, similarly to IL-17A (36). IL-17A and IL-17F m RNA expression were significantly reduced *in vivo* after rapamycin treatment.

Although we did not directly test this, rapamycin may suppress inflammation by promoting autophagy of misfolded HLA-B27. HLA-B27 heavy chain misfolding has been postulated to play a role in SpA pathogenesis (37, 38). In a HLA-B27 transgenic rat model (33-3 rats), rapamycin promoted autophagy-mediated degradation of misfolded B27 heavy chains *in vitro* and could thereby suppress the IL-23/IL-17 pathway (39–41). The presence of HLA-B27 misfolding and the effect of rapamycin treatment on autophagy remains to be tested in our model (21–3 × 283–2 rats).

Rapamycin may also have a direct effect on bone differentiation via mTOR's interaction with several anabolic bone pathways (19, 42, 43). mTOR is a kinase that forms mTOR complex 1 (mTORC1) and mTOR complex 2 (mTORC2) (19), and these complexes integrate signals from a multitude of signaling pathways, including Wnt, PI3K-Akt, IGF, Notch, BMP, and mechanical stress (19, 44–52). Prolonged treatment with rapamycin has been demonstrated to inhibit both mTORC1 and mTORC2 (53), and likely alters bone pathway signaling. The exact mechanism of new bone formation in SpA remains to be elucidated (10, 54). In another model of ectopic bone formation, heterotopic ossification (HO), rapamycin treatment reduced ectopic bone by 50% (55). How the pathways involved in heterotopic ossification (HO) compare to those of SpA may be of interest.

In addition to bone precursor cells and osteoblasts, osteoclasts are also important cellular players in bone remodeling. We did not address osteoclasts in the present study, as the effect of rapamycin on osteoclasts has been studied previously. Inhibition of mTOR has been demonstrated to halt osteoclastogenesis (56, 57) and to improve joint erosions in a TNF-transgenic mice model of rheumatoid arthritis (33). These findings are in line with the attenuated bone erosions we observe in the HLA-B27 tg rats after rapamycin treatment, which could be explained by the inhibitory effect of rapamycin on IL-17A and TNFα production, as both these cytokines promote osteoclastogenesis (58–61).

Given that there is currently limited therapy in SpA, these results may support efforts to evaluate the efficacy of rapamycin treatment for SpA patients. Rapamycin has also been found to reduce inflammation in animal models of psoriasis (62) and colitis (18, 63). These disease manifestations can co-occur with SpA and have overlapping disease mechanisms with SpA (1). The side-effects of rapamycin have been characterized and include hyperlipidemia and osteonecrosis (64). Recently, low dose of rapamycin has been demonstrated to be effcacious in reducing musculoskeletal manifestations in mildly active SLE patients without serious side-effects (65). It is also promising that short-term side-effects such as dyslipidemia may subside after long-term mTOR blocking therapy (64). Moreover, there are new generation small molecules that target the mTOR pathway with potentially fewer side effects. The upstream PI3K/Akt/mTOR pathway may also present an additional set of potential targets for modulating mTOR activity in SpA pathology.

## Conclusions

We provide a rationale for targeting the mTOR pathway in spondyloarthtritis by demonstrating that mTOR blockade with rapamycin inhibits IL-17A and TNFα production by SpA PBMCs and osteoblastic differentiation of human SpA FLS *in vitro*. In the HLA-B27 transgenic rat model of SpA, mTOR blockade reduces arthritis and spondylitis development and severity with decreased inflammation and bone defects with suppression of IL-17A. These results may support efforts to evaluate the efficacy of targeting the mTOR pathway in SpA patients.

## Data Availability Statement

The datasets generated for this study are available on request to the corresponding author.

## Ethics Statement

The studies involving human participants were reviewed and approved by the Ethics Committee of the Amsterdam University Medical Center, University of Amsterdam, the Netherlands. The patients/participants provided their written informed consent to participate in this study. Animal experiments were approved by the Amsterdam University Medical Center (AUMC) Animal Care and Use Committee.

## Author Contributions

SC, LD, and DB contributed to study design, data collection, analysis, interpretation, and wrote the manuscript. MT, VK, LK, LB, and DP contributed to data collection, analysis, and interpretation and revised the manuscript. JT, EG, and MS contributed to analysis, interpretation of the data and critically revised the manuscript. All authors read and approved the submitted version of the manuscript.

### Conflict of Interest

DB is an employee of UCB. The remaining authors declare that the research was conducted in the absence of any commercial or financial relationships that could be construed as a potential conflict of interest.

## Supplementary Material

Supplemental Figure 1 Viability of PBMCs (*n* = 3) in the presence of vehicle (DMSO) and rapamycin. **(A)** FACs gating strategy for DAPI and PI staining. **(B,C)** The percentage of viable cells are shown, normalized to (untreated) control conditions after 48 h of culture (mean ± SD, *n* = 3).

Supplemental Figure 2 The effect of rapamycin on IL-17A and TNFα protein production by synovial mononuclear cells (SFMCs) from SpA patients (*n* = 2) *in vitro*. **(A)** IL-17A and **(B)** TNFα protein concentrations were measured in the supernatant.

Supplemental Figure 3 Viability of fibroblast-like synoviocytes (FLS) in the presence of vehicle (DMSO) and rapamycin (5 nM). **(A)** Measurements by WST-1 assay after 30 min and **(B)** after 2.5 h (mean ± SD, *n* = 4). **(C)** The percentage of Trypan Blue-negative cells after 48 h of culture (mean ± SD, *n* = 3). ns, not significant.
