# Supplementary figures and images for: mTOR Blockade by Rapamycin in Spondyloarthritis: Impact on Inflammation and New Bone Formation in vitro and in vivo
Source: Front Immunol. 2020 Feb 27;10:2344. doi: 10.3389/fimmu.2019.02344 (PMC7065603; doi:10.3389/fimmu.2019.02344)

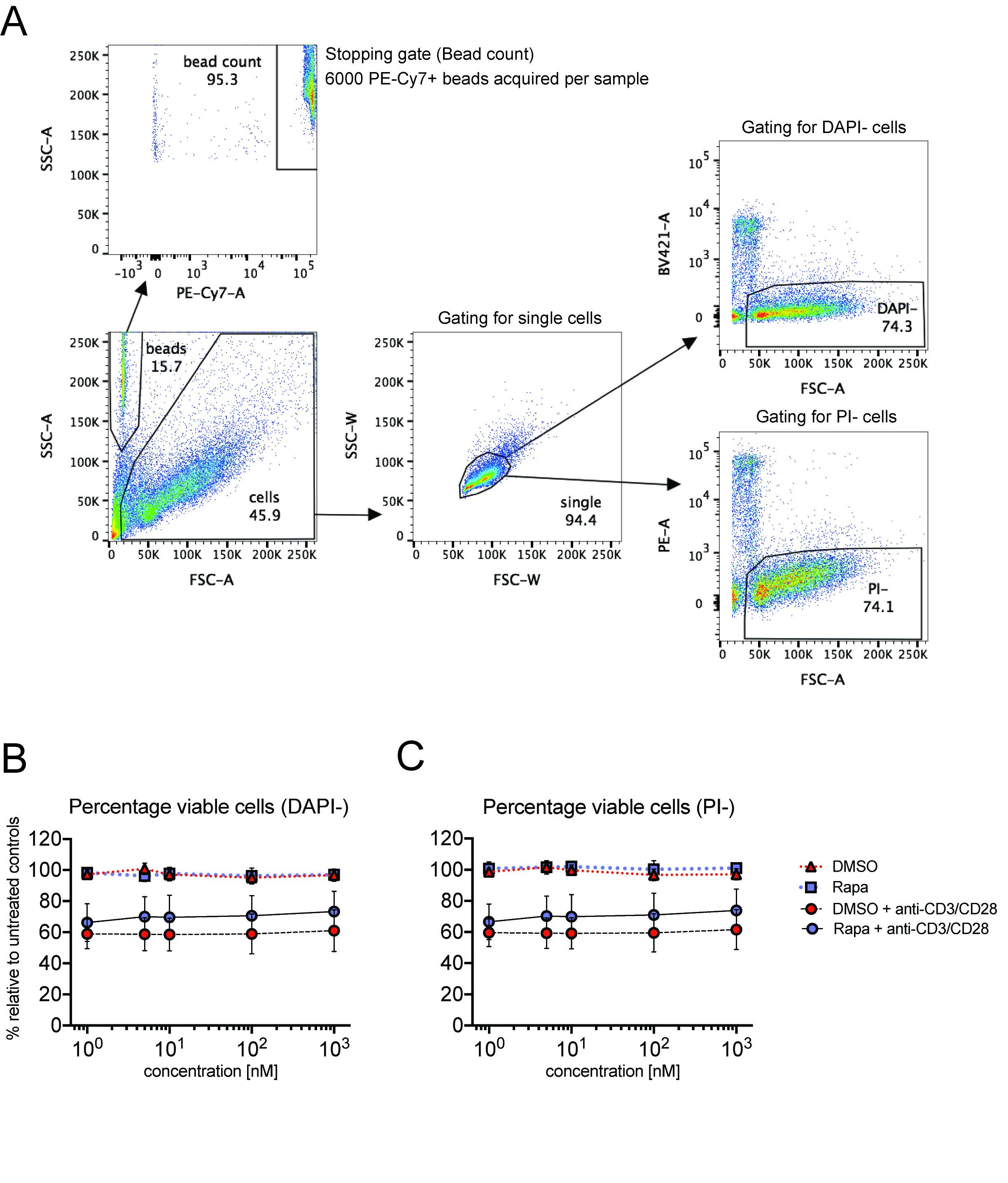

Supplement: Supplemental Figure 1 — Viability of PBMCs (n = 3) in the presence of vehicle (DMSO) and rapamycin. (A) FACs gating strategy for DAPI and PI staining. (B,C) The percentage of viable cells are shown, normalized to (untreated) control conditions after 48 h of culture (mean ± SD, n = 3). [file Image_1.tif]

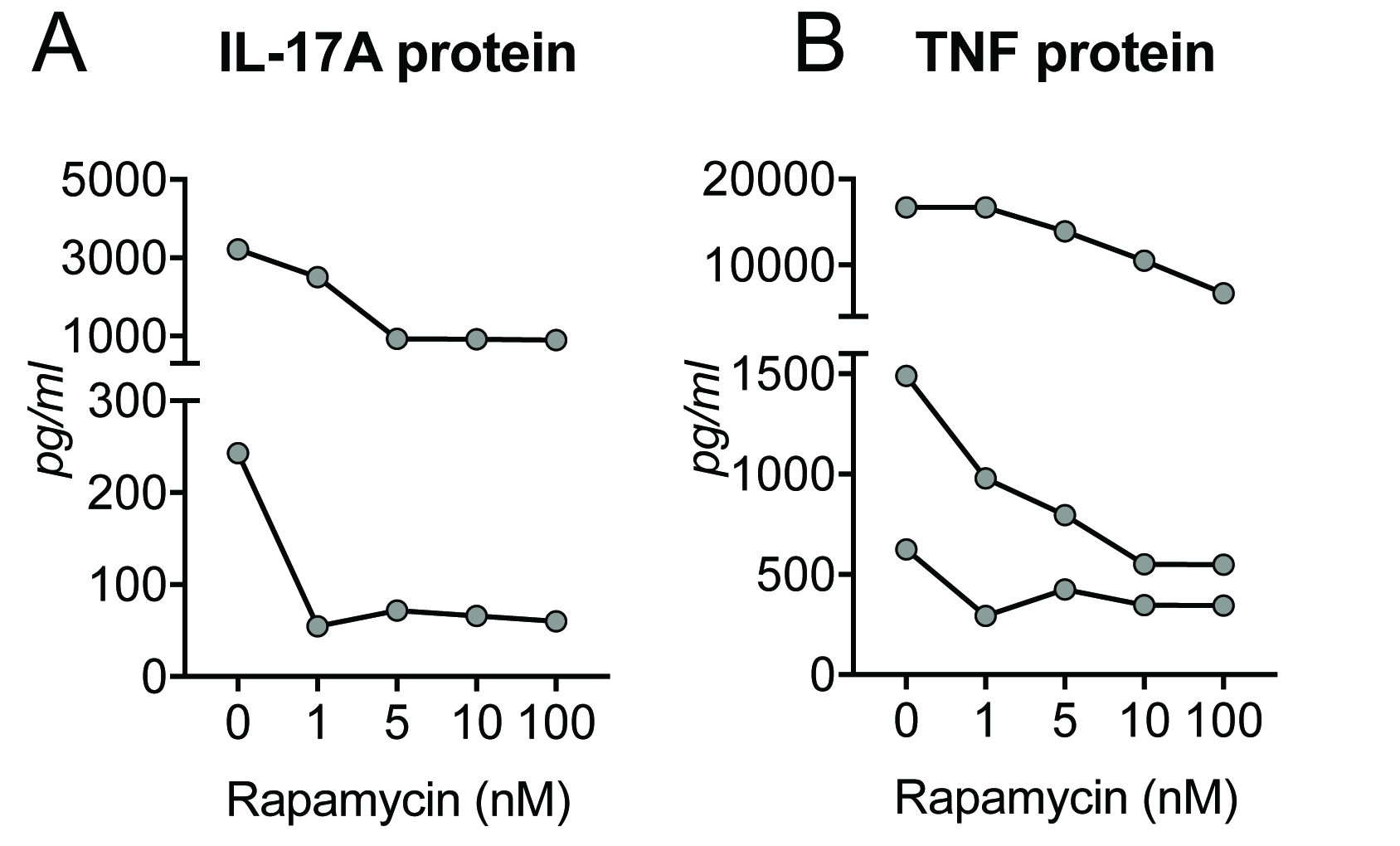

Supplement: Supplemental Figure 2 — The effect of rapamycin on IL-17A and TNFα protein production by synovial mononuclear cells (SFMCs) from SpA patients (n = 2) in vitro. (A) IL-17A and (B) TNFα protein concentrations were measured in the supernatant. [file Image_2.tif]

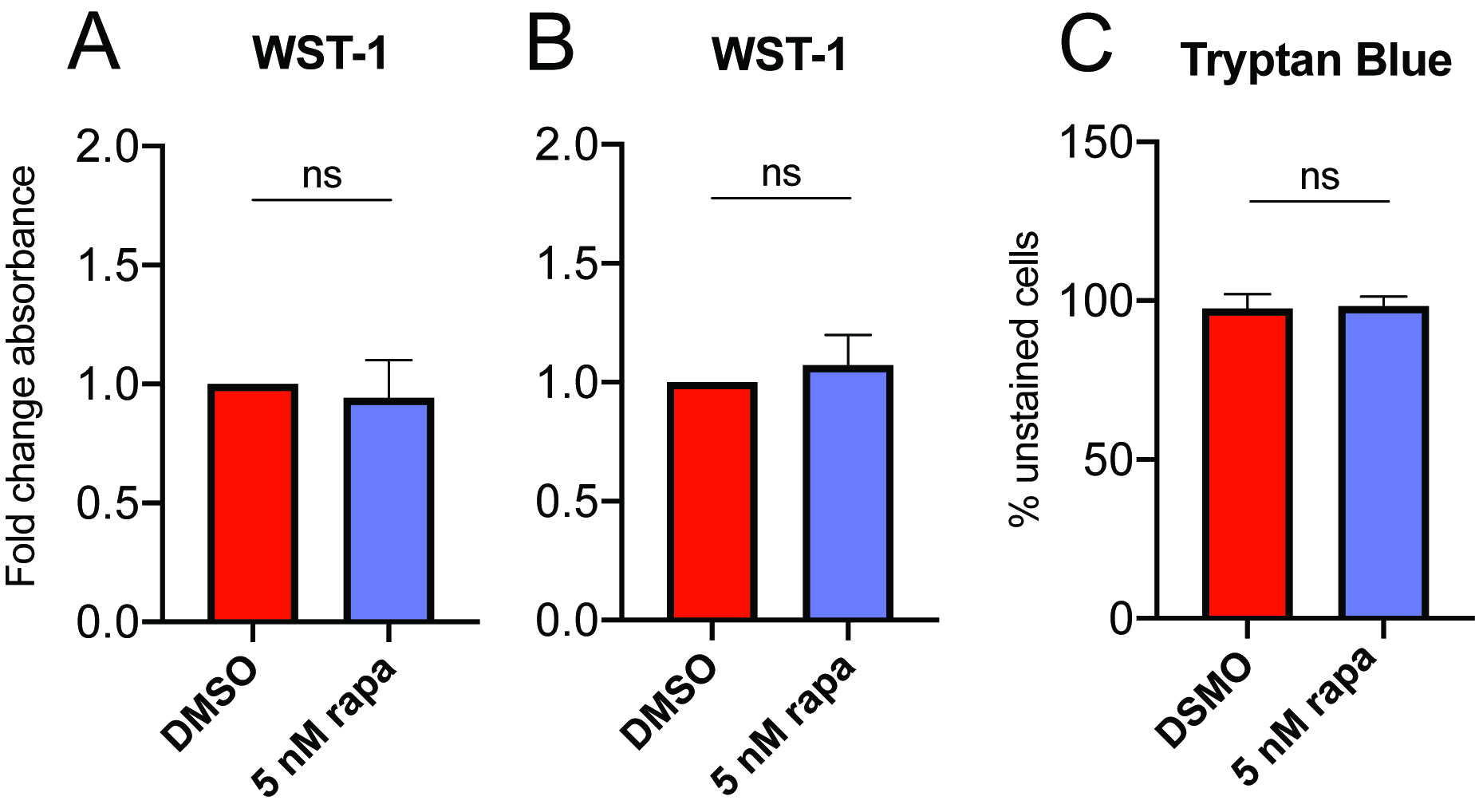

Supplement: Supplemental Figure 3 — Viability of fibroblast-like synoviocytes (FLS) in the presence of vehicle (DMSO) and rapamycin (5 nM). (A) Measurements by WST-1 assay after 30 min and (B) after 2.5 h (mean ± SD, n = 4). (C) The percentage of Trypan Blue-negative cells after 48 h of culture (mean ± SD, n = 3). ns, not significant. [file Image_3.tif]
